# Insights to Study, Understand and Manage Extruded Dry Pet Food Palatability

**DOI:** 10.3390/ani14071095

**Published:** 2024-04-03

**Authors:** Gautier Le Guillas, Pascal Vanacker, Christian Salles, Hélène Labouré

**Affiliations:** 1 Centre R&D Nestlé S.A.S., F-80800 Aubigny, France; gautier.leguillas@rd.nestle.com (G.L.G.);; 2 Centre des Sciences du Goût et de l’Alimentation, CNRS, INRAE, Institut Agro, Université de Bourgogne, F-21000 Dijon, France

**Keywords:** palatability, pet food, dry dog food, dry cat food, extrusion, preference

## Abstract

**Simple Summary:**

Palatability is a pivotal aspect in the fast-growing pet food industry, particularly for dry pet food, which is generally less appealing to pets than wet pet food. As such, research is being conducted to gain a deeper understanding of the acceptability and preference of dry pet food among pets, as well as the impact of the manufacturing process and ingredients used. This review discusses the importance of palatability in feeding companion animals, the various ways of assessing acceptance and preference, and the potential influence of both internal and external factors on the palatability of products. Additionally, knowledge on common and novel ingredients as well as process parameters is presented, with the aim to offer a better understanding of palatability management.

**Abstract:**

Pet food production is a fast-growing industry. While extruded dry pet food is the favored pet food due to its convenience of use, it may have poorer palatability than other pet foods such as wet pet foods. However, palatability plays a pivotal role in meeting nutritional requirements or providing therapeutic benefits in cats and dogs, as it ensures food acceptance. Thus, both academics and manufacturers conduct routine palatability tests to assess acceptance and preference of products among pets, alongside sensory analyses involving human panels. Palatability is greatly influenced by species-specific and environmental factors in cats and dogs. The review will hence present the current knowledge on palatability assessment and animal food perception; it will then aim to explore strategies for effectively managing palatability in dry pet food by examining the impact of key ingredients and process parameters on the finished product’s palatability. Moreover, the demands and needs for sustainable and healthier products as well as supply constraints present novel challenges and opportunities for academics and manufacturers.

## 1. Introduction

The pet food market has been steadily growing over the past few years, reaching more than 120 billion U.S. dollars of sales worldwide in 2022, exhibiting an increase of 20 billion dollars since 2019 [1]. This represents over 35 million metric tons of global production worldwide, and Europe and North America are the main contributors, with more than 11 million metric tons each [1]. With 150 pet food companies established in Europe, employing approximately 118,000 people in 2022, the industry experiences tough competition to feed the estimated 340 million pets of Europe [2]. Cats and dogs are favored companion animals, with 127 and 104 million individuals, respectively, in 2022 for Europe [2]. The history of this flourishing industry dates back to the 1870s when James Spratt introduced the first dog biscuits in the U.K. [3]. Since then, pet food science, technology, and safety have not stopped evolving to offer a wider range of nutritionally complete and balanced products [3,4]. However, palatability, i.e., the ability of a food, based on its sensory characteristics, to be accepted and consumed by pets, is essential. Pet food can be categorized into three formats depending on their final water content: dry (less than 14%), semi-moist (14 to 60%), and moist (more than 60%) [4,5]. While moist foods, e.g., canned food and pouches, appear to be preferred by cats and dogs [6], dry pet food remains the favorite choice of pet owners due to its convenience in terms of storing and feeding [5].

Assessing the acceptance and preference of a food by validated methodologies such as one-bowl tests for assessing acceptance and paired tests for assessing preference [7] is of high importance. Properly evaluating palatability is the best way to ensure that pets will receive their daily nutritional requirements by consuming their entire meal and not rejecting it [7]. By focusing on evolution, genes, and dietary needs, preference trends might be observed, such as a high-protein diet for cats due to their obligate carnivore regime [8] and a greater preference for sweet or higher tolerance for starch-rich products by dogs due to their omnivorous diet requirements [9,10]. Academics and manufacturers addressed the issue of preference in cats and dogs by evaluating their ability to perceive taste, odor, and mouthfeel [10,11,12]. These species-specific factors may also be influenced by external components such as socialization or the environment [13,14].

Regarding the product itself, managing dry food palatability will require an optimal blend of ingredients coupled with an adequate process. Dry pet food is composed of sources of carbohydrates (such as cereals), sources of proteins (such as meat, fresh or frozen), byproducts, or meals, and sources of lipids from animal or vegetal origins. Their origins as well as the way they are incorporated (i.e., concentration, mixed, or coated) will affect the product’s palatability [15]. In addition, their selection must meet nutritional and palatability requirements but is also influenced by regulations, availability, cost, trends, and pet owner’s beliefs. Sustainability is a new dimension that focuses much research on new ingredients such as agricultural byproducts or alternative sources such as algae and insects [16]. For the last 70 years, dry pet food has essentially been produced by extrusion cooking, giving it a specific aerated texture [17]. To obtain such products, ingredients endure high temperatures and high pressure that can strongly affect either positively (e.g., flavor generation) or negatively (e.g., destruction of nutritive constituents, poor texturization) their physicochemical characteristics and potentially palatability [17,18].

**Figure 1:**
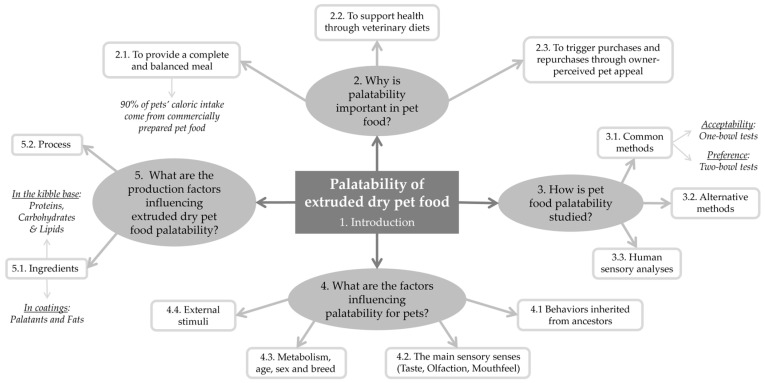
Mind map presenting the questions discussed in this review.

Therefore, this review will provide insights into the importance of dry pet food palatability and the reasons why it should be investigated. It will be followed by an overview of how to assess palatability and the diverse factors that may influence cat and dog perceptions of products. This review will then focus on ingredients and processes that impact palatability. Figure 1 visually summarizes the different questions raised in this review.

## 2. Providing Palatable Meals: A Challenge for Pet Food Manufacturers

Manufacturers have the responsibility to provide nutritious meals that will be consumed by cats and dogs. Thus, they have to respond to their specific needs, depending on their age, reproductive status, or medical condition [19,20], as well as being sufficiently palatable to ensure the ingestion of the daily nutritional and energy requirements. Manufacturers must challenge themselves to produce pet food that is as palatable as it is nutritious while convincing pet owners of the quality of their products.

### 2.1. Complete and Balanced Meals for Everyday Life

Unlike feral animals, which feed through hunting, foraging, or scavenging to meet their nutritional needs [21], companion animals only have access to what the owners provide, resulting in a poorly diversified range of foods until the quantity by container or storing unit is finished. Aside from some treats, snacks, or scraps offered by owners, a large majority of pets are fed with commercially prepared pet food, representing up to 90% of their caloric intake in developed regions or countries such as North America, Northern Europe, Japan, Australia, and New Zealand [6,22]. A survey dating from 2019 on 2181 pet owners in the U.S. emphasized that 89% of them fed commercially prepared food to their pets and that more than half of the respondents would primarily feed their pets with dry food only [23]. In contrast, in an Italian 2020 survey of 914 pet owners, 65.3% of them would buy both dry and wet pet food, and 24.7% would buy only dry food [24]. This discrepancy might be explained by the cultural differences between North America and Europe in terms of the relationship between pet owners and their animals as well as their place in society, as highlighted by Lowrey [25].

Commercially prepared dry food is highly convenient for pet owners due to its relatively long shelf life, and ease of storage and feeding to pets [6] as presented and compared with the other range of pet foods in Table 1. However, it hinders cats and dogs from reaching their nutritional needs by varying their meals the way feral animals might do. Consequently, it obliges manufacturers to produce complete and balanced products to offer cats and dogs their daily specific nutritional needs in one single food that is palatable enough to not be rejected by the pets. To meet nutritional guidelines, manufacturers can rely on recommendations from several independent bodies such as the National Research Council (NRC), the Association of American Feed Control Officials (AAFCO), and the European Pet Food Industry Federation (FEDIAF) [26]. It can thus be challenging for manufacturers to meet the requirements of high-nutritional-quality food while staying palatable [20,27]. As stated by Becques et al. [27], “It does not matter how well-formulated a diet [is] if [pets] will not eat it”.

### 2.2. Supporting Health with Veterinary Diets

Good palatability not only facilitates the ingestion of all needed nutrients in a meal, but it also plays a major role in more specific veterinary (therapeutic) diets [32]. Two main concerns might appear in this case: veterinary products are often less palatable, and ill pets may have a reduced appetite [20,33,34].

Veterinary foods appear to be less palatable than regular food because their formulation is specialized for nutritional or therapeutic needs. Bourgeois et al. [32] emphasized, for instance, that the restricted amount of fat and protein or the presence of components with unpleasant taste in veterinary products (such as a stronger bitterness of low-molecular-weight added peptides) can make it challenging to obtain a palatable dry food. Indeed, fats are known to be highly palatable, while bitterness, as identified by humans, is mostly rejected by animals [35,36]. However, regarding cats, masking this taste with sweet-tasting compounds, which is often done for humans, would not reduce the bitterness, as cats do not taste sweetness (discussed in Section 4.2.1) [8].

The lower palatability of therapeutic diets might also amplify the reduced appetite of sick pets. Resulting from their state of health (cancer, anorexia, etc.) [20,33], appetite loss may distress owners who will look for alternative commercially available dry products that might appear more palatable but are consequently less nutritionally adapted to their pets’ condition [20,31,37].

It is hence of great interest for manufacturers to better understand palatability drivers and adapt their formulations to increase the palatability of veterinary food [34]. Many studies have been recently conducted on new ingredients or formulations for their health-beneficial effects (such as insect-, plant-, and algal-based meals or byproducts) looking at their impact on pet conditions (weight, digestion, cardiology, immunity) and palatability, offering new opportunities in pet food nutrition and potentially the veterinary pet food range [38,39,40,41].

### 2.3. Owner-Perceived Pet Appeal Leading Purchases

Similar to the parent–baby dynamic, a pet owner will have to interpret their pet’s behavior toward the palatability of a dry product and determine whether the pet likes it or not [42]. Pet caretakers interpret their cat or dog’s responses by paying attention to specific behaviors before, during, and after meals, such as excitement or attention during the preparation of the bowl, latency time between the bowl being offered and the moment the pet starts eating, speed of consumption, etc. [27,43,44].

Between the wide variety of pet foods present in the marketplace and the numerous distribution channels available, such as supermarkets, pet stores, veterinarian offices, and e-commerce, pet owners have the opportunity to be highly selective when purchasing dry food for their companion animals. Owner-perceived pet appeal is therefore considered by manufacturers as an important parameter for the purchase of their products by pet owners [45,46]. Indeed, according to the survey conducted on 935 Italian pet owners by Vinassa et al. [24], palatability is considered one of the top priorities when choosing a pet food with a score of 4.2/5 on a scale from 1 = not important to 5 = fundamental. Similarly, for Americans surveyed by Schleicher et al. [23], pet preference was sixth of 24 criteria with a grade of more than 3.5 on a scale from 1 = not at all important to 5 = extremely important. On the other hand, once the purchase is triggered, the manufacturers must ensure repurchase by also making their products acceptable enough for the owners. For that matter, both palatability testing and sensory analyses on pet food can then be conducted to evaluate preference or acceptance by pets and humans [42,45]. Pet food producers must combine in their products high nutritional value, high palatability for animals, and acceptance by pet owners to achieve customer loyalty [34,45,46].

## 3. Evaluation of Pet Food Palatability

Palatability “is related to how readily a food is accepted and measured in terms of its attractiveness and consumption” [47]. For pets, this attractiveness is mainly led by the overall flavor of the product, defined in humans as the combination of “tastes, smells, trigeminal, and tactile sensations as well as the visual and auditory cues, that we perceive when tasting food” [48]. As human-to-animal extrapolation is an arduous and hazardous task in terms of perception and pleasantness, researchers and manufacturers have implemented commonly used assays to evaluate either the acceptability or preference of dry food by pets. Alternative methodologies and even human sensory evaluations can provide new insights into the palatability of pet food.

### 3.1. Common Methods

**Figure 2:**
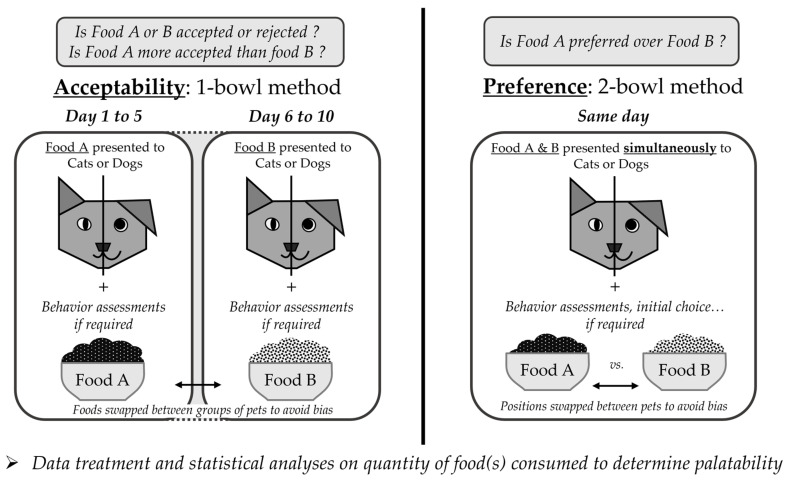
Common methods to evaluate pet food palatability.

Common methods to evaluate acceptability and preference for dry pet food have been discussed by several authors in books and reviews [42,46,47,49]. A presentation of the common methodologies to assess acceptability and preference is summarized in this section and in Figure 2.

#### 3.1.1. Acceptability: One-Bowl Method

The one-bowl, single-bowl, or one-pan test is a monadic feeding test that corresponds to offering free access to only one product at a time to a pet for a defined time. They are often repeated for five days or longer, not only to alleviate the potential impact of neophilia or neophobia, i.e., love or fear for a new or novel product but also to give time for caloric adjustment by the pets [42,49]. Food intake is evaluated by weighing the difference between the food being offered and the food remaining in the bowl at the end of the test. Overall, acceptability is defined by the amount of food consumed, allowing sufficient caloric intake [42]. Nevertheless, when comparing the acceptability of two distinct products (for example, food A and food B) by only considering the amount consumed, a notable increase or decrease might appear over time due to the novelty effect or caloric adjustments of the second food tested by the cats or dogs. Therefore, a ratio of the total food A divided by the sum of total food A and food B consumed during the test period might offer a clearer response to determine which food was more accepted. Hence, a ratio of 0.50 would indicate a similar acceptance of both products [42,49]. However, when comparing products with very different caloric values, the latter must be taken into consideration when interpreting one-bowl tests because caloric adjustment will impact volume intake [49]. Similar to neophilia, the anti-apostatic effect has to be considered when data are interpreted. This effect refers to animals that consume more of something known but rarely available than a similar palatability product that is commonly served. Indeed, it has been noticed that cats with a rich past feeding experience tended to eat more of the rarer kibbles when presented against more common kibbles with similar palatability. However, this effect is yet unknown in dogs [32,50].

Moreover, monadic tests enable the assessment of eating behaviors as a response to the food offered, such as speed of consumption and enthusiasm for eating the food or turning it down [27,47,49]. For that purpose, and the fact that it does not require extensive or specific training for the pets compared to other feeding tests, one-bowl tests are commonly chosen for in-home-use-tests (IHUTs) that include detailed questionnaires regarding both owners’ acceptability and perception of the acceptability of the product by their pets [42,47,51]. While IHUTs might be more representative of the real use of the products, its “uncontrolled” environment and possible subjective interpretation by pet owners may lead to variations in results. Tobie et al. [47] recommend that IHUTs be performed on approximately one hundred individuals when thirty are sufficient to offer robust results for expert panels in kennels or catteries. Nevertheless, discrepancies between in-kennel and in-home dogs’ palatability results due to different experiences or living environments can still be noted. Indeed, Griffin et al. [13,49] showed that in-home dogs were more stable in their preferences, while in-kennel expert dogs were able to better discriminate slight palatability differences in products.

Tobie et al. [47] concluded their review by summarizing that the one-bowl test is a valuable method to validate a product in development, mimic acceptability under in-home eating conditions, obtain owner perception during IHUTs, observe eating behavior, and to gain knowledge on the nutritional aspects of the food or compare highly different products. One-bowl tests are effective for determining the degree of acceptability of a product but do not provide preference data [42].

#### 3.1.2. Preference: Two-Bowl Method

When benchmarking or developing new products, manufacturers want to ensure that their new products offer at least a similar or higher palatability than competitors or their previous products. In that aspect, two-bowl tests (or “two-pan”, “versus”, “paired”) are widely used and aim to compare the consumption of two dry foods (for example, food A and food B) in two different bowls that are offered simultaneously to the pets [42,46,47,49]. The two foods are presented for a limited amount of time (15 to 30 min for dogs, up to 24 h for cats) or until the pet has consumed the equivalent of its recommended caloric intake to avoid overconsumption [42,49]. The tests are often repeated with the bowl position switched to alleviate side or position bias, which reflects a preference for the pet to choose first the left or right bowl [47]. Foods are weighed before and after the tests to evaluate the quantity of products consumed. The food with the higher consumption could be interpreted as the preferred food. Griffin [49] recommended considering results as an intake ratio, being the proportion of one food consumed over the sum of the proportion of each of the two foods consumed (A/(A + B)). This offers an “objective, reliable and [mostly] bias-free” response to the two-bowl method. Moreover, when comparing more than two foods, it is sometimes interesting to rank them while maintaining the ease of the two-bowl test conditions. For that purpose, Rogues et al. [52] presented the Bradley-Terry model, a commonly used statistical model to analyze paired comparison data. The model enables one to rank several products by estimating their percentage of winning against another product.

Another interesting parameter to observe is the initial choice, or first choice, which is the first bowl out of which the pet will take a bite or eat a minimum of a certain weight of food and reflects olfactory attractiveness [46,47]. A strong initial choice is often correlated with a higher food intake [53,54,55].

In conclusion, two-bowl tests are useful for developing new products and/or detecting fine differences of preference (due to changes in formulation or process, benchmarking), but this comparison between two products presented simultaneously would not represent common eating behavior for pets at home [47]. Offering the two products simultaneously enables a more accurate evaluation of the small differences in palatability than one-bowl tests. Moreover, as highlighted by Samant et al. [46] in their review, despite their reliability, versus tests “tend not to be sensitive to long-term satiating effects of food while accounting for nutritional and caloric value”. Two-bowl tests are one-shot tests or repeated once, and cats and dogs do not have the time to make caloric adjustments to the tested foods. They also emphasized the complexity and the increasing numbers of required two-bowl tests when more than two products are compared as well as conclusions on preference when two products show no difference in intake ratio and first choice.

### 3.2. Alternative Methods

To gain insight into the reasons for a non-preference between two different foods during two-bowl tests, Basque et al. [56] completed them with an olfactory discrimination test. Expert dogs were presented with two odorant stimuli of the tested foods through a specifically designed two-port canine olfactometer to evaluate their capability to discriminate the odors. Dogs, trained to recognize a specific food odor to earn a treat, would sniff at each port and stop at the one they believed to be delivering the proper odor. By this means, they were able to conclude that dogs could differentiate two products by olfaction while showing no preference for one or the other despite different odors.

Nonetheless, the use of such an apparatus requires extensive training [53,56]. Hence, methods have been tested to evaluate the efficacy of untrained, or less trained, dog panels while relying on dogs’ strong sense of smell. Hall et al. [53] and Thompson et al. [57] successfully proved that non-consummatory tests could accurately predict preference in a population of dogs with only a short training. In the Hall et al. [53] study, two different products were presented to dogs under a removable fiberglass screen, allowing them to see and smell the foods. After 15 s of exploration, the screen was removed. The food chosen first was the most consumed 89% of the time, suggesting that dogs do not need to taste food to make a choice. Similarly, Thompson et al. [57] correlated the most consumed food among two products to the one for which the dogs showed the most interest when presented in two separate bowls under grids (time spent investigating Food A versus time investigating Food B). For years, olfaction has been at the center of many studies for dogs’ preference as a key driver in food selection. In that respect, Pétel et al. [54] designed false-bottom bowls to add odorant compounds in a compartment under the food. Two-bowl tests with this device, comparing the same food but different odorants led the dogs to choose first and consume more of the food covering the most palatable odorant.

Tobie et al. [47] and Rogues et al. [52] presented complementary methods and approaches focusing on behavior such as kinetics (speed of eating, number of meals, etc.), exploratory behavior and body language (role of olfaction, licking and sniffing, etc.), enjoyment (prompted by incentive salience) or even physiological indicators (pets surface temperature, heart rate, etc.) to determine preferences that were applied to either cats or dogs. To the authors’ knowledge, alternative palatability assessments regarding cats focus on behavior more than experiments with specific apparatuses such as the dog olfactometer or false-bottom bowls detailed above for assessments on dogs. For instance, Becques et al. [27] video-recorded cats’ behavior during two days of two-bowl tests comparing a very palatable kibble (VPK) against a low-palatability kibble. They noticed that the cats spent significantly less time sniffing the VPK during the two first visits of the bowl on the first day of the test before eating it, signifying less hesitation to consume this food. Other behaviors, such as licking or speed of consumption, were not affected by the palatability of the tested food.

### 3.3. Sensory Analysis by Human Panels

For human food, sensory analyses on pet food can be either descriptive (description of one or more attributes or characteristics on a scale) or discriminative (perception of differences or not between two or more products) and are mostly led by expert, trained panelists [45,46,58]. Studies conducted at Kansas State University widely used descriptive analyses to evaluate the impact of process or specific ingredient inclusion on product specificities and quality [55,58,59,60,61]. Among their research, Di Donfrancesco et al. [58] developed a lexicon for the sensory properties of dry dog food. It provided more than 70 descriptors, or attributes, to evaluate flavor, aroma, texture, and appearance. To obtain such results, five highly trained panelists evaluated 21 U.S. dry dog foods and established as exhaustive a list as possible of both common and specific attributes to offer a number of descriptors. Despite obvious physiological differences regarding taste and flavor perception between humans and cats or dogs, sensory analyses conducted on human beings provide valuable information on product characteristics. Coupled with palatability tests on pets and physicochemical analyses, such as gas chromatography and mass spectrometry for aroma or compression and penetration for texture, some attributes driving preference or rejection in pets can be estimated [45,58].

## 4. Internal and External Factors Influencing Flavor Perception and Food Consumption

### 4.1. Behaviors Inherited from Ancestors

Domestic cats (*Felis catus)* and dogs (*Canis lupus familiaris*) are classified within the mammalian order Carnivora. This taxonomic group encompasses a diverse range of animals that share a common ancestor that underwent evolutionary divergence in various lineages. Contrary to its name, Carnivora comprises not only obligate carnivores such as the Felidae family (including cats) but also herbivorous species such as pandas (only herbivorous in Ursidae) and red pandas (Ailuridae), frugivorous animals such as kinkajous (Procyonidae), and omnivorous animals such as the Canidae family. Within the Canidae family, certain members exhibit a predominantly insectivorous diet (e.g., certain fennecs), while others rely on fruits and plants when prey become scarcer (e.g., some coyotes) [21].

Domestic cats are part of the Felidae family and are obligate carnivorous. Descending from *Felis silvestris lybica*, the North African wildcat, with domestication potentially dating up to 9500 years ago, although strong evidence from ancient Egyptian art presents domestication at 4000 years B.P. Recent studies have proved potential domestication, or at least a mutualistic relationship with humans, at approximately 5500 years B.P. in China [62,63]. This domestication, compared to that of dogs, see below, resulted in smaller changes between domestic cats and their wild counterparts, *Felis silvestris*. As solitary hunters, Felidae cannot attack large prey the way a wolf pack can. For that matter, they are known to multiply hunt sessions targeting smaller animals. According to Fitzgerald and Turner [64], cats typically hunt approximately 12 small prey as rodents to meet their daily energy and nutrient requirements. Even domestic cats fed with commercially prepared food do in fact divide their daily meals into smaller portions throughout the day [27,32,52], reflecting their inherent hunting heritage.

Domestic dogs are parts of the Canidae family and, therefore, are omnivorous, indicating their ability to feed on food from both meat and vegetal origins [21,35]. They are known to descend from wolves, *Canis lupus*, and are recognized as one of the first domesticated animals, with domestication going back from 100,000 to 14,000 years ago [21]. The feeding behavior and palatability attributes of dogs may be influenced by their evolutionary history from wolves. *C. lupus*, as pack hunters, has the capacity to take down large mammals, a rich source of protein and fat. However, such kills are not a daily occurrence, and the pack leader, or alpha, has priority access to the food, leaving juveniles to compete for leftovers. This hierarchical behavior and limited access to food during spaced out hunts, coupled with scavenging in the early stages of domestication, may explain the observed tendency of today’s domestic dogs to rapidly consume large quantities of food. The process of domestication has also strongly impacted the behavior of dogs, as feral dogs now primarily rely on scavenging a broad range of food sources, including fruits, plants, insects, animal carrions, and even feces, rather than engaging in active hunting [21,42]. Moreover, probably due to this long domestication process and modified eating behavior, dog metabolism has also been impacted, giving them an enhanced adaptation to a starch-rich diet compared to their ancestors (wolves, *Canis lupus*) [9], which did not occur in cats [64].

### 4.2. Insights and Roles of the Main Sensory Senses Involved in Flavor Perception for Pets

#### 4.2.1. Taste

In both species, these ancestral inherited habits and dietary regimes are closely related to their sensory perception and metabolism. Cats’ and dogs’ sense of taste is adapted to a carnivore pattern. Both companion animals possess a majority of taste receptors that respond to “sweet” amino acids, as perceived by humans, such as L-proline and L-cysteine. While these taste receptors are also activated by mono- and disaccharides in dogs, they are completely insensitive to sugars in cats [21,35,36]. To date, cats are considered entirely insensitive to sugars, which is an uncommon trait among mammals. Conversely, dogs are known to have a sweet tooth [7]. These taste receptors are also specifically inhibited in cats by “bitter” amino acids, such as L-tryptophan, which are rejected by cats [65].

As other bitter compounds are often encountered in plants as an indicator of toxicity, carnivorous animals are less likely to include such substances in their diet. Moreover, there are fewer or limited-function T2R genes for bitter taste receptors in carnivores than in other animal groups. Indeed, the proportion of noncoding genes increases from dogs (omnivores) to cats (obligate carnivores), with only 15% of pseudogenes in dogs and 40% in cats, despite the same number of T2R genes in the two species [66].

Both animals also possess acid-sensitive and nucleotide-sensitive taste receptor units, with the latter associated with an “umami taste”. Dogs possess few receptors of “fruity-sweet” compounds while cats do not. Instead, cats have receptors for more bitter compounds, such as quinine, tannic acid, and alkaloids. These discrepancies between cats and dogs are congruent with the obligate carnivorous diet of cats, allowing for a more refined appreciation of amino acids from meat without interference from sugars [21,35]. Indeed, while dogs possess the sweet taste receptor heterodimer TAS1R2 + TAS1R3, cats do not have the capacity to encode the TAS1R2 protein, resulting in the loss of the sweet taste receptor [36,67]. There is a debated hypothesis on the absence of sweet taste receptors in some Carnivora that relates it to specific feeding specializations such as obligate carnivore. Indeed, this feeding specialization would have no benefit from having such a taste receptor [68,69]. Recently, Wolsan and Sato [67] supported this hypothesis by predicting the presence or absence of the sweet taste receptor using data on genetics and behavioral taste preference tests. Carnivora with an obligate carnivore diet studied here, such as cats, all lost their sweet taste receptors, with few exceptions for which the loss is hypothesized to be ongoing.

#### 4.2.2. Olfaction

Animals, especially dogs, are thought to have sharper olfactory capabilities than humans [70]. Indeed, many studies on dogs have shown their very low detection threshold capacities for chemical substances in medical areas and disease detection, reaching a concentration of one part per trillion [71,72]. As enumerated by Hall et al. [70], dog olfaction is also useful for law enforcement for narcotics, explosive detection, or even matching suspects. However, Laska [71], Wackermannová et al. [73], and McGann [74] all highlighted in their reviews the growing debate on whether human olfaction is genuinely weaker than that of other mammals. They emphasized the lack of congruency between methodologies employed between species, making comparison difficult, and a nonsignificant number of subjects or odorants tested.

Concerning palatability, olfaction is considered essential in food selection and preference for cats and dogs [7,33,36,42,56,67,75]. Regarding its physiology, the mammalian olfactory system is similar for most species, including these two companion animals. The mammalian olfactory system is composed of two main parts: the main olfactory epithelium in the nasal cavity, stimulated by the orthonasal pathway, e.g., when smelling or sniffing food, or by the retronasal pathway, e.g., mostly during food consumption and mastication, and the vomeronasal organ above the palate in the mouth, primarily stimulated by pheromones and low-volatiles compounds [72].

Cats are selective animals concerning their meal, and their sense of smell is a great tool to evaluate the edibility and potential palatability of the food [36,42]. As stated by Aldrich and Koppel [42], the cat olfactory system is finely tuned to detect unfamiliar odors, leading to a thorough assessment of food freshness and safety. In a previous study, Mugford [75] was able to increase the voluntary intake of 20 cats by blowing “attractive” food odors over their diet, emphasizing the hedonic impact of food aromas on consumption.

Olfaction is also of course primordial in food choice and palatability for dogs [21,54,76]. When presenting several bowls of food, the first choice ends up being strongly correlated with the intake ratio, suggesting a positive impact of odor on food choice and consumption [53,56]. This is also confirmed by the fact that dogs appear to make their choice based on the odor given off by the food even though they have no access to the food [53]. For their part, Pétel et al. [54] adapted the two-bowl methodology by creating false-bottom bowls containing an olfactory stimulus in the compartment beneath the foods. The exact same dry food was offered to dogs in the two bowls, but different olfactory cues were placed beneath it. Overall, the most palatable scent led the dogs to significantly choose the food in the bowl above it. Despite the undeniable driving power of olfaction in food choice for dogs [54], it is only a part of the sensory food experience. Dogs are indeed able to discriminate between odor A and odor B without showing any preferences between food A and food B [56], suggesting that olfaction is not the only parameter in palatability.

Moreover, Zoon et al. [77] highlighted how olfactory cues could impact food choice and how this food’s taste was congruent with the presented odor. They further extended this congruency in odor/taste to odor/energy density. By making dogs smell olfactory cues recalling high- or low-energy density products, they were able to increase their appetite for the matching energy-density product.

#### 4.2.3. Mouthfeel

Other senses might also impact palatability as the overall mouthfeel of food. Textural properties, size, shape of the kibbles, or even meal temperature are known to affect the preference of cats and dogs [11,33,35,78]. This is particularly true for cats, as they tend to take small bites and take more time eating than dogs [35]. Therefore, mouthfeel can play an important role in palatability for cats, especially those suffering from anorexia. Michel [78] suggested that even a “subtle change in kibble shape or food consistency could have a profound impact on a cat’s acceptance of a new food”. In the case of dogs, Sagols et al. [12] demonstrated that dogs weighing less than 10 kg did not show a significant preference between cross-shaped or round-shaped kibbles, while dogs with a body weight above 10 kg consumed significantly more cross kibbles. It is noteworthy that the two populations received a different diet based on their size, which may have influenced the results. However, some manufacturers patented specific kibble shapes for dogs, leading to different eating behaviors (time of ingestion, prehension) [79,80]. Despite there being no palatability tests cited in the patents, it is possible to hypothesize that different eating behaviors may induce different preferences.

### 4.3. Internal Factors Impacting Food Ingestion: Rapid Insight into Metabolism, Age, Sex and Breed

Looking back at the definition of palatability, it can be defined “as the momentary and subjective orosensory pleasantness of food consumption […]; however, in the long-term, the perceived palatability of a food is strongly influenced by its post-ingestion consequences, and this effect can override sensory factors” [81]. Post-ingestion outcomes are some of the many non-sensory-related internal factors influencing food choice. While the bulking effect of food in the stomach is probably a limiting factor in food consumption [49,75], meeting nutrient requirements appears to be driving the eating experience of cats and dogs [82]. When simultaneously offered a variety of differently formulated products and after prior experience with the foods, the two species will adapt their intake ratio to meet their nutrient and energy requirements in terms of protein, fat, and carbohydrate ratio, apparently despite product palatability [8,82,83]. The ability to self-regulate macronutrient intake was also confirmed by Hall et al. [81]; however, different macronutrient ratios were observed due to their different methodologies to balance product palatability. Therefore, previous eating experience can impact palatability testing if not correctly planned. Specific palatability protocols are then necessary to prevent biased results. However, it is notable that nutrient needs, sensory abilities, and behavior differ depending on age, sex, reproductive status (neutered or intact), and breed [72,81,84,85]. Hall et al. [81] noticed that consumption of calories from protein would differ in cats or dogs according to their age and fat or lean body. A regression tree analysis demonstrated that cats with the lowest lean body mass tended to consume more calories from protein, while this is also true for dogs with the highest fat body mass. Age may also affect appetite as well as sensory performances; for example, Chan et al. [84] emphasized reduced sensory-specific satiety in older dogs as in humans, while Pekel et al. [36] and Kokocińska-Kusiak et al. [72] stated a degeneration of olfactory and taste capacities in aged cats and aged dogs. These studies also reported that sex differences may be observed with a higher activity of olfactory cells in female dogs. However, Alegría-Morán et al. [86] showed no differences in food preference between male and female dogs but mentioned previous studies [87,88] that compared intact males to neutered females, suggesting a higher impact of the animal’s reproductive status. Finally, despite the lack of clarity of breed effects on palatability factors, morphological and physiological discrepancies observed in the olfactory system between brachycephalic or dolichocephalic breeds may impact aroma perception and therefore food preferences [85].

### 4.4. Additional Factors: Impact of External Stimuli

As with all animals, cats and dogs have cognitive abilities and can be influenced by their own experience or their social and external environment. Flavor and nutritive experiences were discussed in previous paragraphs with the phenomenon of neophilia/neophobia or regulation of nutrient intake, but pets are also able to remember unpleasant experiences. Bradshaw [10] related studies on food aversion tests by adding lithium chloride to dog or cat foods. He characterized dogs as “slow to learn and quick to forget”, while cats would rapidly and for a long time refuse the food, even if the same food, but unaltered, was presented. Cats appear to be more sensitive to aversion, and therefore, one bad experience may result in long-term rejection.

Pets, mostly dogs, are well known to create a strong bond with their “human parent”, and the owner’s behavior may influence the behavior of their dogs toward food choice [89]. Instinctively, dogs would choose a bowl with a large quantity of food versus a bowl with a small quantity when there is no interaction with the owner. However, they will significantly show a preference for the small quantity when the owner expresses interest in this bowl. The trend for choosing the bowl for which the owner shows interest is even stronger when the choice is between two equally small quantities of food [89]. This corroborates with the results from Griffin et al. [13] showing discrepancies in preferences between in-kennel dogs and in-home dogs, indicating that owners may influence the choices of their dogs.

Living with conspecifics can also impact feeding behavior. Mugford [75] reported a phenomenon known as social facilitation of feeding in puppies, wherein food consumption increases in group settings compared to when they are alone. However, this behavior was not observed in adult cats or dogs. Additionally, he [75] highlighted that agonistic behaviors were scarcer in both cats and dogs when food was consistently accessible, as opposed to when it was limited to specific mealtimes. In her later review, Finka [14] confirmed “increased frequencies of affiliative behaviors” between cats with greater food availability. Therefore, dominance plays an important role in the hierarchization of groups when resources are limited [75,90]. Lupfer-Johnson and Ross [91] demonstrated that interactions between dogs can also induce preference. They showed that dogs would consume more of a product previously eaten by a conspecific after scenting its breath.

Weather and seasonality can also influence food consumption [72,86,92]. Weather and air humidity may improve olfactory skills when humidity is increased by increasing odor intensity. In contrast, reduced humidity, coupled with high temperature, can lead to dehydration of the nasal cavity, resulting in lower olfactory efficiency [72]. Alegría-Morán et al. [86,92] found that seasons also impact food intake in both cats and dogs. Pets appeared to eat more in cold seasons (autumn and winter) compared to hot seasons (spring and summer). However, they noticed that the food intake would start to decrease as the body weight increased, resulting in a higher food intake in autumn than in winter. This is interpreted as an adjustment of caloric intake to counterbalance heat loss during cold seasons. Nevertheless, no impact of seasonality was demonstrated on food preference for either species [86,92].

Cats and dogs are living beings with specific physiological, morphological, and cognitive characteristics inherited or acquired throughout life. Adding to that their interactions with their environment and their ability to socialize, there are an uncountable number of influences that can impact food intake and food preference. Therefore, manufacturers need to take into consideration these factors when formulating, producing, and testing their products.

## 5. Managing and Improving Dry Pet Food Palatability

### 5.1. Formulating and Choosing Ingredients to Modulate Palatability

#### 5.1.1. In the Kibble Base

All ingredients included in a pet food are chosen for a specific purpose. Some of the primary interests of ingredients in the kibble base are of course to meet nutritional requirements in terms of nutrient composition and digestibility and to offer proper functionality during the extrusion cooking process. In a more practical way, manufacturers also consider their availability, cost, and sustainability. However, the palatability of these ingredients is also considered to produce appealing food for pets [15,27,93].

As mentioned previously, cats and dogs possess specific macronutrient requirements that must be fulfilled in terms of proteins, carbohydrates, and fats. The palatability of the final product is greatly influenced by the origin of these nutrients.

Proteins are principally brought by meat, meat byproducts, and meals (e.g., beef, pork, poultry) [15,93]. However, for cost reasons, availability [94,95], or for increasing concerns regarding global warming considerations [96], alternative protein sources or specific diets [93,96] are already used, such as plants (e.g., corn gluten meal, soybean meal), or are emerging, such as insects (e.g., black soldier fly larvae, yellow mealworm, adult house cricket…). Despite offering new possibilities, they also add new challenges in terms of palatability [15,96,97].

Plants, such as grains, root vegetables, legumes, and fruits, are the main source of carbohydrates. Corn, wheat, rice, or oats will be the most common ones, as rich sources of starch that will also impact the structure of the kibbles during the expansion, giving it their specific porous structure [93,96,98]. Fruits and vegetables will also provide fiber [98].

Lipids originate from either animals or plants. They can be added per se or as part of the previously cited ingredients, such as meats and meat byproducts or oleaginous plants [99,100]. Similar to starch, fats can greatly influence the structure of the kibble by reducing the dough viscosity, resulting in lower expansion.

##### Protein Sources

The main sources of protein in pet foods derived from animals encompass a variety of meats, such as beef, poultry, lamb, or pork, either under raw conditions or as byproducts. According to the European Pet Food Industry Federation (FEDIAF), meat refers to skeletal muscles, while byproducts are defined as “entire bodies or parts of animals, products of animal origin or other products obtained from animals, which are not intended for human consumption”. This includes organs (e.g., heart, liver, lungs), blood, carcasses, skins, feathers, and more [101,102].

Palatability might be dependent on the animal species of the meat, the type of meat, e.g., muscles, organs, or other byproducts, and its condition, whether it is unprocessed or in a processed meal form. In the early eighties, Houpt et al. [7,76] demonstrated that dogs could express a preference for food smelling like meat, and, more precisely, they preferred pork or beef over lamb or horse. More recently, Samant et al. [46] also considered the overall raw meaty flavor desirable to cats and dogs in their review, however, without exhibiting a preference for a specific species. In fact, Funaba et al. [103] showed similar acceptance between meat meal (species not mentioned, but usually beef) and chicken meal in cats. This demonstrates that cats would accept each protein source tested here equally if presented separately. Nevertheless, there seems to be a limited understanding of the preferences that pets may have for certain species of meat over others [104]. This lack of knowledge may be attributed to factors such as the methodologies used to assess preferences, the manufacturing process of pet food, pet personal individuality, and previous experiences. All of these parameters can significantly influence the perceived preferences of pets [105,106].

From the pet owners’ perspective, fresh meat benefits from a more healthy, nutritive, and palatable image than meals and byproducts [107,108], but it does not reflect cat and dog food preferences. Koppel et al. [109] compared the impact of the inclusion of mechanically deboned chicken, i.e., fresh poultry, against the inclusion of chicken byproduct meal on the sensory characteristics and volatile profile of kibbles. The volatile compound concentration slightly increased with the inclusion of fresh material, but only small differences were shown by the descriptive sensory analysis between the two products. Over the 30 descriptors evaluated by human panelists on appearance, taste and aftertaste, flavor, texture, and mouthfeel, only bitterness, fishiness, and cohesiveness of mass were affected by the inclusion of fresh meat over meat meal. Recently, some studies compared fresh meat or chicken to meat meals and meat or chicken byproduct meals [107,108]. In both studies, two-bowl tests showed a significant preference for byproduct meals over fresh materials, with an intake ratio and first choice of approximately 70% for the processed ingredients. Meineri et al. [107] attributed this preference to the higher fat content and moisture in kibbles with byproduct meal and its lower drying temperature, which may have impacted aroma generation, thus influencing first choice and overall palatability. However, in a study by Shields [108], the fat content was slightly lower in byproduct foods, and it was highlighted that a higher fat content could lead to undesirable higher lipid oxidation. The author hypothesized that aroma strongly impacted palatability. Similar to what Koppel et al. [109] mentioned, volatile analysis showed a higher concentration of compounds in a fresh chicken-containing recipe than in kibble with chicken byproduct. It should be noted that in both studies [108,109], less favored products, i.e., those with fresh meat, released lower concentrations of Maillard reaction products, which some are known to be highly palatable [46], and higher concentrations of lipid oxidation compounds, such as aldehydes, resulting in rancid aromas [108] that are potentially linked with depressed palatability. Additionally, a hedonic test on kibbles from Shields [108] showed that human consumers preferred the aroma of the product with chicken byproduct meal and assumed that dogs would favor it too. However, the human panelists changed their minds once made aware of the protein source to prefer fresh chicken food. Overall, byproducts seem to be favored over fresh ingredients by pets, but the reason is still under discussion.

Despite the increase in fish use in the pet food market [110,111], relatively few studies can be found on their impact on palatability when included in dry pet food. However, fish components such as salmon protein hydrolysate proved to be a potential interesting ingredient for improving dry dog food palatability [112].

In recent years, a trend to humanize our relationship with our four-paw companion animals has led to an increased demand for alternative foods with a lower carbon footprint impact and protein sources that align with owners’ beliefs. Additionally, meat proteins, especially beef, have been shown to be the principal source of food allergies in cats and dogs [113,114,115]. For these matters, the inclusion of plants as a protein source has proven to offer comparable digestibility to animal-derived protein despite reluctance from some researchers focusing on animal nutrition; for example, vegan diets would not meet the nutritional needs of pets, especially cats, as obligate carnivores [85]. Nonetheless, the palatability of plant-sourced proteins compared to that of animal-based proteins has been questioned.

Knight and Satchell [85] surveyed more than 4000 dog and cat owners on the perceived palatability of vegan diets against conventional diets. The survey showed that, from the owners’ point of view, vegan pet food appeared to be at least as palatable as conventional pet food.

From a technical palatability point of view, only a few studies have focused on the palatability of plant-based kibble compared to that of animal-based kibble and vice versa. Ambiguous results were found regarding whether plant-sourced proteins would be preferred over animal-sourced proteins by pets. While several studies [46,76,116] assessed a preference for animal-based food by cats and dogs, the methodology and overall recipes were questioned by Callon et al. [117]. These authors conducted palatability tests by the one-bowl methodology coupled with behavioral observations to assess preference between dog kibbles with animal- or vegetable-based ingredients as the main source of protein. Yet both kibbles contained chicken palatant and chicken fat, the latest being in higher concentration in vegetable-based kibbles to achieve similar fat content in both recipes. While no differences were noticed in terms of “feeding rate, level of distraction, hesitation, or anticipatory behaviors between the two diets”, expressing a possible similar palatability, they observed higher post-consumption interest (licking of bowls and remaining focused on bowls) for meat-based kibbles, thus demonstrating a higher palatability of the meat-based diet in dogs. Acknowledging the higher concentration of poultry fat in the plant-based diet, which probably led to increased palatability and feeding rate, the researchers hypothesized that the higher post-consumption interest for the animal-based diet was due to the lower satiety effect of animal proteins. Recently, new sources of plant-based protein have been considered, such as distillers dried grains with solublesor corn-fermented proteins. These protein sources are highly nutritional, and they also proved to be palatable to cats, possibly due to the presence of yeast, known for its high presence of nucleotides appreciated by cats [118].

Insects are a new trend for protein sources in both human and pet foods. Insects proved to be highly nutritional and sustainable [97,119,120], but questions remain concerning palatability. An olfactory attractiveness study of different species of whole air-dried insects against dry dog food as a control emphasized the potential interest of insects for dogs. Gender preferences were noted, with males being more attracted by mealworms while females being more attracted by Turkestan cockroaches [121]. However, acceptance in dry pet food appears to be species- and inclusion rate-dependent [97]. Bosch and Swanson [97] reviewed that dogs would accept food containing black soldier fly larvae (BSFL) meal and banded cricket up to 10% and 24%, respectively, while only up to 5% BSFL oil in the coating was accepted. Regarding cats, they would accept only BSFL meal or oil up to 5% in the food and BSFL oil up to 2.5% in the coating [97]. Despite increased interest in insects as valuable protein sources, published studies on their impact on palatability in dry pet food remain scarce [97,122,123].

##### Carbohydrate Sources

Carbohydrates are often viewed negatively by pet owners, as they believe they are not digested by cats or dogs [93]. Their known evolutionary pathway from wolves or wild African cats and comparison with today’s wild counterparts reinforce this meat-eater image [124]. Thus, carbohydrates, and mostly grains, are sometimes considered “fillers” and thus unnecessary by pet owners in pet food [15,93]. However, it is incorrect to call these ingredients “fillers”. As stated by Thompson [15], “There is no room in a pet food formula for any ingredient that is added just to fill space”. Carbohydrates are of source important for texturization of the kibble during the extrusion process due to the starch content, but starch, once metabolized, is also an important source of glucose for cellular energy. Additionally, carbohydrates are sources of dietary fiber for proper intestinal health and satiety [15,93,98].

As an important part of the formulation, e.g., from 30% to 60% in dry dog food and up to 35% in cat food [125], the impact of carbohydrates on palatability must not be neglected. The source of carbohydrates will significantly impact end-product characteristics. Koppel et al. [126] showed that grain-free dog kibbles were less aromatic than grain-added recipes, which presented a higher concentration of volatiles, primarily aldehydes. The higher concentration of aldehydes in grain-added products correlated with a rancid aroma was attributed to cereals, as it is commonly present in such ingredients. However, this study also emphasized the complexity of dry dog food aroma, which was probably also influenced by other ingredients, as all products studied in this context had different sources of proteins [126]. Further studies are therefore needed to better evaluate the effects of carbohydrate sources on palatability.

Limited research has been conducted on the impact of different sources of starch on the palatability of dry pet food [127]. Previous studies have primarily focused on the effects of extrusion parameters and starch concentration on product quality, as starch plays a crucial role in structuring during the manufacturing process. This will be discussed later in this review.

However, Li et al. [127] demonstrated that corn starch in baked treats was favored over whole wheat flour and was significantly more palatable than tapioca flour, potato flour, or chickpea flour (in the order mentioned). The preference for cereal-based starches is hypothesized to be due to their potentially higher aromatic strength, as mentioned by Koppel et al. [126]. Nevertheless, it is important to note that the absence of meat in the treats and the different processes (baked vs. extruded) introduce notable discrepancies between the two studies. This made it difficult to draw definitive conclusions regarding the positive impact of corn starch on palatability for dogs, and further research is needed. Indeed, it was found that increasing the concentration of potato starch (PS) from 0 to 300 g/kg at the expense of corn in extruded dry puppy food enhanced palatability compared to corn alone [128]. Due to the different amylopectin/amylose ratios in starch between the two ingredients, adjustments in extrusion parameters, such as water addition and drying temperature, were necessary, which significantly influenced the characteristics of the final products. Higher levels of potato starch resulted in increased expansion and hardness of the kibbles, as well as decreased density. Texture, particularly increased hardness, and reduced density with the addition of PS, was identified as a potential factor influencing the intake ratio. Additionally, the moisture content of the finished product was highlighted as a crucial feature in palatability, with higher moisture levels being preferred, as assessed by de Brito et al. [129]. Interestingly, a product with 300 g PS/kg exhibited similar palatability to one with 0 g PS/kg, despite having 2% lower moisture. However, when moisture levels were equalized between the two kibbles, the palatability of the product with 300 g PS/kg significantly increased, highlighting the enhancing effect of moisture. However, while there is an undeniable positive influence of PS and moisture on palatability, the method used to equalize the moisture level is not mentioned, and the physical characteristics of kibbles were not analyzed afterward. Therefore, the higher palatability of PS may not solely be attributed to initial texture differentiation, and further investigation is needed.

In 2013, De Godoy et al. [130] stated that dietary fibers gained increasing interest in the pet food industry for their beneficial effect on digestive health, but limited scientific information was available, especially in terms of palatability. Beet pulp and cellulose have been commonly used in pet food [130,131,132,133,134], and new sources of dietary fiber are considered for their nutritional benefits, but their palatability interest remains to be determined. To explore new sources of fiber, researchers and manufacturers have focused on discarded byproducts to develop sustainable and value-added ingredients for pet food [55,131,132,135,136]. The inclusion of dietary fiber, its origin, and its concentration may affect both texture and flavor and, consequently, palatability [131,135,136]. Sugarcane fibers (9% inclusion) and wheat bran fibers (32% inclusion) were significantly rejected by dogs compared to a control diet without fiber inclusions. This might be attributed to the higher bitterness perceived by a human sensory panel in kibbles containing sugarcane fibers [55]. Souza et al. [131,132] showed the ability of fiber inclusion to reduce product density in comparison to a control without included fibers, with an impact of the source and the inclusion rate. Cellulose led to the lightest density compared to cassava fiber and beet pulp. Increasing cassava fiber inclusion from 4% to 12% increased the product friability and expansion index. Contrary to sugarcane and wheat bran fibers [55], cassava fibers (12% inclusion), cellulose, and beet pulp in extruded dry dog food led systematically to a higher intake ratio when tested against a control without included dietary fibers, and cassava fibers had a stronger positive effect. In contrast, citrus fruit-sourced fibers such as citrus pulp pellets and orange pulp resulted in higher density, lower expansion, and higher hardness when included in dry pet food [135,136]. However, orange pulp increased the palatability, and citrus pulp pellets resulted in similar palatability when both were included at 6% and compared to a control without fiber inclusion [136]. These studies may emphasize the potential higher impact of flavor (aroma and taste) over texture on palatability for dogs, as, despite opposite texturization, fiber inclusion resulted in similar increased palatability [55,131,132,135,136].

Regarding cats, Donadelli et al. [133,137] and Finet et al. [138] studied the inclusion of Miscanthus grass as a dietary fiber source. Contrary to the previous ingredients presented, Miscanthus grass is produced for its fiber content and hence is not labeled a “byproduct”, which may be favored by pet owners. The inclusion of Miscanthus grass had a limited impact on the product process and physical characteristics when included at 9% [138] or 10% [137] and did not impact food intake when compared to control products and beet pulp or cellulose-containing products for similar total dietary fiber (TDF) content [138]. In contrast, soybean hulls appeared to have a higher food intake than beet pulp products when included at similar TDF content [139]. Beet pulp kibbles exhibited a higher rejection from the cats. Each fiber source has a different composition [137], especially in terms of TDF content. Aiming for similar end TDF in the product thus implies different concentrations of ingredients. As a result, this may impact end-product characteristics differently, which may have induced higher or lower intake ratios. Previous studies showed no effect of beet pulp on the intake ratio when added up to a level of 12.5% inclusion, while, in [139], its content reached an amount of 15.5%, which may potentially explain its higher refusal.

As the main ingredients in dry pet foods, sources of carbohydrates must be carefully selected. Despite their origin and inclusion rate, their effect on final product physical and chemical properties may significantly influence cat and dog acceptability or preference. Moreover, not only will they structure and aromatize the kibble, but diverse sources of carbohydrates will also offer a wide range of essential nutritional components (e.g., vitamins, fatty acids, minerals, amino acids, etc.) [140,141].

##### Lipid Sources

Lipids, along with proteins and carbohydrates, are essential macronutrients that play crucial roles in providing a balanced and complete diet for companion animals. Among these nutrients, lipids are the most calorie-dense, offering 9 kcal/g (37 kJ/g) of metabolizable energy, while carbohydrates and proteins provide only 4 kcal/g (17 kJ/g) of metabolizable energy each. Despite their reported high palatability, it is important to moderate lipid intake. Lipids are also important for their composition in essential fatty acids. As previously mentioned, lipids are derived from complex ingredients such as meat, fish, and oleaginous plants, but they can also be added separately to the kibble base during extrusion. However, fats and oils are predominantly coated on kibble to maximize their palatant power. Moreover, extrusion can negatively affect fats and oils through lipid oxidation [142], resulting in potentially higher rejection by pets and pet owners due to their specific aroma [108,143].

In-kibble fat has seldom been studied for its palatability effect rather than its nutritional contributions. Basal fats can influence perceived palatability due to their energy density, as cats and dogs are able to adapt their energy intake [8,81,83]. It has been shown that cats consume less of a high-fat product than of a low-fat product to achieve the same gross energy intake with both recipes when presented separately, despite similar palatability in two-bowl tests [144].

To overcome the potential negative effect of fats and oils during extrusion, especially on expansion, Kim et al. [145,146] investigated the impact of the inclusion of whole soybeans (WSBs) on process and palatability to increase the energy density of dog kibbles due to their high fat and protein content. To incorporate WSBs, levels of corn gluten meal and brewer’s rice in the basal recipe were reduced as well as chicken fat in the coating. Increasing WSB inclusion from 0% to 30% negatively impacted product expansion and density due to the higher fat content in the extrusion, which reduced shearing. Despite a reduced topical chicken fat content on the kibble and textural differences between the products, palatability was not affected. This similar palatability was potentially attributed to the preference for soybean meal over poultry meal, as with the samples used by Félix et al. [147], due to the higher content of low-molecular-weight sugars in soybean meal and the higher total dietary fiber content in WSBs, which may be rejected by dogs [55]. However, the authors did not acknowledge the 3% moisture difference between the two products (0% and 30% WSB inclusion), which has also proven to be an important factor in palatability [128,129,148].

Currently, insects are also considered alternative lipid sources in pet food [123]. No negative effect was observed when vegetal oil was completely replaced with insect oil in broilers, offering promising prospects for pets [149]. In fact, black soldier fly larvae oil inclusion at 2.5% and 5.0% proved to be equally accepted by dogs in dry food without physiological impact [150]. However, relatively limited research has been conducted on the palatability of insect oil-based diets for cats [143].

Ingredients in pet food are selected based on various criteria, including their nutrition, functional aspects, and palatability. Ingredients can serve as sources of various nutrients that can influence palatability in multiple ways. The complexity of extruded dry pet food recipes poses a challenge in evaluating the impact of each ingredient on overall palatability. With new consumer trends and the introduction of alternative ingredients to the market, the possibilities and challenges in the field of pet food continue to expand.

#### 5.1.2. In the Coating

Coating is the final step of extruded dry pet food production before packaging. Dry pet food is coated for multiple reasons. The coating will protect the core from oxidation, humidity, and friability and provide complementary nutrition to the kibble, but it is mostly the primary way in which to increase palatability. Since the coating is ultimately on top of the product, it diffuses an attractive smell and is the first taste perceived by the animal before mastication [151]. Dry pet food will preferentially have multiple layers encompassing fats and palatants. Fats can be of either animal or plant origin. Palatants can be liquid or solid (powder) and are generally mixtures of animal or vegetal tissues as partially hydrolyzed (chemically or enzymatically) protein sources [46,152,153,154].

##### Fats and Oils

The main fat sources used in coating dry pet foods are poultry, pork, and beef. Fournier [155] compared 11 types of coating fat from the three abovementioned origins and identified a significant effect of their origin on dogs. According to her work, a mix of pork and beef fat would be the most palatable choice, while poultry would be less palatable than pork alone. Recently, Inal et al. [156] showed higher palatability of sunflower oil over poultry fat or beef tallow when coated on dry dog food. Similar results were reported for cats [33]. Inal et al. [156] imputed the greater preference for sunflower oil to its higher linoleic acid concentration and hypothesized the importance of specific fatty acid composition on palatability. In fact, it appears to be fundamental when looking at managing palatability. MacDonald et al. [157] reported in their review that medium-chain triglycerides (MCTs) enriched in the medium-chain fatty acid (MCFA) caprylic acid (C8) depressed cat acceptance, while enriched lauric acid (C12) levels did not. Dhakal and Aldrich [158] observed similar results in dogs in that when applying three MCFAs, caproic acid (C6), enanthic acid (C7), and caprylic acid (C8), on kibbles, a palatant would be needed to mask their aroma to sustain effective palatability. Subsequently, fatty acid chain length could be considered a factor influencing appreciation by pets. Manufacturers have focused on fat fractions and fatty acid ratios to enhance palatability for cats and dogs. In the same way as MacDonald et al. [157], patent U.S. 11,510,423 B2 [159] asserts that specific weight ratios of medium-chain fatty acids C12:00/C10:00 (from 0.85 to 2.5) and/or C14:00/C12:00 (from 0.45 to 4.3) would improve palatability when coated on dog or cat kibbles.

In another patent application, Lin [160] provided a method to produce a processed coating mixture containing at least 80% by weight of animal fat combined with diverse flavor precursors. They underlined the drastic content loss in short- (SCFA) (from C2 to C5) and medium-chain fatty acids (from C6 to C10) in their processed composition in comparison to unprocessed fats. A quantitative descriptive human sensory analysis comparing processed coating mixtures of pork fat or chicken fat to the unprocessed fats highlighted a reduction in animalic, pungent, and sour notes attributed to the lower SCFA content. Two-bowl tests on 25 dogs (processed vs. unprocessed pork fat) and 25 cats (processed vs. unprocessed chicken fat) emphasized the palatability-enhancing effect of the processed coating mixtures.

Externally applied fat can also provide nutritional benefits as basal lipids. In patent application WO 2020/123965 A1 [161], polyunsaturated fatty acid (PUFA) sources as algal oils are considered for their docosahexaenoic acid (DHA) content. This PUFA is especially important for brain function. The invention discloses a refinement method of *Schizochytrium*, *Aurantiochytrium*, or *Thraustochytrium* algal oil to increase their palatability by at least 20 to 45% in comparison to crude algal oil. Two-bowl tests conducted on dry dog food coated with algal oil at different steps of the process demonstrated an increase of more than 50% in the intake ratio (from 22.9% to 74%) against dry dog food coated with fish oil. The aforementioned steps comprised a degumming and/or a refining step by using a short-path evaporator and a deodorizing step. A deodorizing step was essential to significantly reduce burnt, fishy, green, and chemical/solvent notes from the previous steps but also reduced the offensive flavor intensity fivefold. Deodorization somehow lessened some lipid oxidation products (1-penten-3-one, 4-heptenal and 2,6-nonadienal) but drastically decreased Maillard reaction products and other volatiles (trimethylpyrazine, 2-ethyl-3,5-dimethylpyrazine, 2-ethyl-3,6-dimethylpyrazine, 2-hydroxy-3-methyl-2-cyclopenten-1-one, methyl-1H-pyrrole-2-carboxaldehyde, and indole) by a factor of 100–1000 down to barely detectable levels, especially pyrazines, which were present at very high contents in crude algal oil. For similar lipid oxidation volatile concentrations, palatability increased with the incremental loss of the selected Maillard reaction products.

Therefore, processing fats and oils may enable more palatable and more nutritionally adequate food for animal companions.

##### Palatability Enhancers

Animal or vegetal hydrolysates, either in liquid or dried powder form, are common palatants in the pet food industry and are complex hydrolyzed mixtures of animal or vegetal tissues sometimes garmented with flavor precursors and other palatable compounds. Animal digests are the main palatants in the pet food industry. They often comprise non-rendered animal byproducts such as organs, viscera, fat, and meals and contribute to the meaty flavor. When exposed to heat, physical shearing, acids, and/or enzymes (chemical and/or enzymatic hydrolysis), the ingredients are reduced to a mixture of short-chain proteins and/or peptides [162,163,164]. During their manufacturing, a Maillard reaction will occur and is sought after for its flavor-adding ability. The Maillard reaction is well known and has been well described in many publications since the beginning of the last century [46,165,166,167]. It is a complex chain reaction initiated by the carbonyl group of e.g., a reducing sugar and an amino compound, e.g., an amino acid, favored by heat and low moisture content, resulting in the generation of flavoring compounds (with a meaty flavor among other aromas) and browning [165,166,167,168]. Added ingredients and production parameters are therefore cautiously chosen to manage this voluntarily generated chemical reaction to obtain the appropriate flavor generation.

Chen et al. [169] focused on the volatile generation produced by the Maillard reaction in seven palatants and their impact on the palatability of dry dog products. Brewer’s yeast, chicken meal, and soybean meal were chosen as the protein sources, and xylose was chosen as the reducing sugar. Process parameters were set as a pH from 6 to 9, and the temperature ranged from 100 to 140 °C. While, unfortunately, no complementary information is given on concentration and process, the seven palatants were revealed to have significantly different palatability and volatile profiles. A total of 23 aroma compounds out of the 53 identified were correlated to palatability, with a confirmed positive impact reported for benzaldehyde, vanillin, and 2,5-dimethylpyrazine.

U.S. 2008/0085350 A1 from Shi et al. [170] demonstrated the palatability-enhancing potential of spiking 2-methyl furan (at 2 ppm or 0.02 ppm), a Maillard reaction product [171], in a composition based on chicken byproduct digestion at 70% and Maillard reaction precursors when used on dry pet food. While the chicken byproduct digest itself was less palatable than the commercial palatant derived from poultry liver, the composition reached similar palatability, and when spiked with 2 or 0.02 ppm 2-methyl furan, it was even more palatable for dogs. More patents or patent applications already described in Samant et al.’s review [46] emphasized the positive impact on both palatability and owner perception of liquid aroma coated within the palatability enhancer or prior to it (US 2015/0056347 A1, U.S. 2016/0309749 A1 and U.S. 6379727 B1). They also propose a list of past patents describing the positive impact of Maillard reaction precursors, such as various reducing sugars (glucose, xylose, fructose, molasses, etc.), free amino acids, sulfur-containing compounds, and diverse animal byproducts.

More recently, in their patent application, Ashie et al. [162] discussed the positive impact of added dairy fat mixed in a liquid animal digest (LAD). Lipolyzed and non-lipolyzed dairy fat were compared in a two-bowl test with 20 dogs. Concentrations of 1 to 3% (by weight) lipolyzed or non-lipolyzed dairy fat in LAD were added to a control pet food without dairy fat. The results showed higher palatability for non-lipolyzed dairy fat than for lipolyzed dairy fat, potentially due to the lower content of free fatty acids (such as SCFAs) generating unpleasant flavors. Coconut oil in LAD at 1% by weight was also tested instead of dairy fat but did not improve palatability.

With increasing concern for the environment and raw material supplies, researchers have looked at novel, sustainable protein sources with lower costs for formulating dog food attractants. In a recent study, mushrooms (*Lentinus edodes*) and mealworms (*Tenebrio molitor*) were considered replacements for chicken liver [172]. Chicken liver-based palatants had a significantly higher ingestion rate (>60%) than both insect- and mushroom-based palatants when tested in the two-bowl test. Differences were also noticed in volatile analyses, with higher importance of sulfur compounds for chicken liver, 1-octen-3-ol for *L. edodes* and pyrazines for mealworms. It is noteworthy that while the dog food attractant was only a hydrolysate of mushrooms and insects, the chicken liver-based attractant also contained Maillard reaction precursors such as xylose as a reducing sugar and glycine and glutamic acid as amino acids. Maillard reaction products are palatable for dogs [46], and this difference between the three recipes may have played an important role in the results of the palatability tests. However, the two novel ingredients reacted in a similar positive manner as chicken liver when tested against uncoated dry dog food. Their palatability could then be improved by the addition of key flavor agents.

Similarly, patent application CN 115088786 A [173] discloses the potential palatability-enhancing ability of three fresh or frozen insects, whole *Tenebrio molitor* worms, whole *Hermetia illucens*, and whole silkworm chrysalises. The palatability mixture was obtained by mixing and emulsifying insects with or without enzymes under specific pH, temperature, and time conditions. Over 10 examples presented in the patent, insects could be mixed with a Maillard reaction precursor system (xylose, glycine, cysteine hydrochloride, glutamic acid, thiamine, and yeast extract) before undergoing a flavor modifying treatment (mix of beta-cyclodextrin and yeast powder), with or without protease. Other tested mixtures could only be mixed insects with the flavor modifying treatment, or with the Maillard reaction precursors. Two other mixtures tested were only mixed insects. Preference and acceptance tests were conducted on dogs. Over the 10 examples, only the five with protease, flavor modifying treatment and Maillard reaction precursors achieved a significant increase in the intake ratio to 80% versus the control. Palatability enhancers containing only the mixed insects were strongly rejected with less than a 15% intake ratio.

Plant-based palatants are also a sustainable alternative to animal digests; however, cats appear to still be reluctant to such palatability enhancers while dogs show mixed results [174].

Several patents or patent applications have reported the positive impact of organic acids when added to coatings, namely, ascorbic, sorbic, succinic, and gallic acids, on palatability for dogs [46] or phosphoric, citric and hexamic acids for cats [175,176]. However, a patent from [177] found that despite its potent palatability-enhancing ability at a very low concentration (0.0016% added on kibble), ascorbic acid did not improve palatability in cats. Given that ascorbic acid is naturally present in fruits and vegetables, it may be hypothesized that cats are less responsive to such compounds due to their strict carnivorous diet, in contrast to dogs, which are omnivores.

### 5.2. Adapting the Process in Favor of Palatability

**Figure 3:**
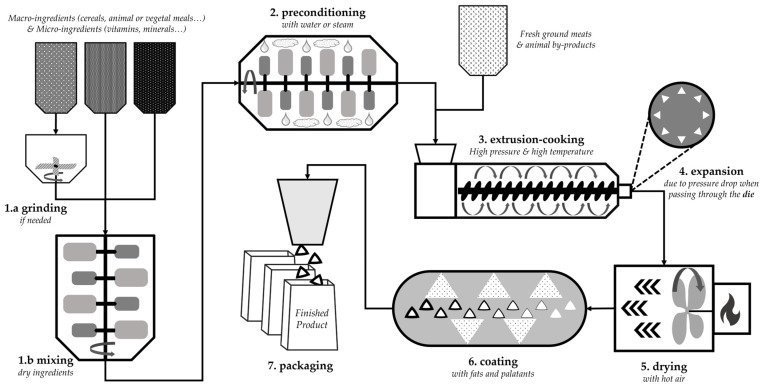
Making of dry pet food by extrusion-cooking. Numbers referring to the process description in Section 5.2.

Extrusion has been the main process used by manufacturers to produce dry pet food since the 1950s [17]. The process (Figure 3) involves several steps, starting with (1) the uniform delivery of dry raw materials previously grounded and mixed to (2) a preconditioner. The preconditioning step mixes, moisturizes, and precooks the dry mix by adding steam and/or hot water to achieve a temperature typically between 70 and 90 °C. The mixture is then transferred to (3) the extruder, where thermal and mechanical energy are applied to the dough under high pressure (ranging from 34 to 37 atmospheres), resulting in molecular changes contributing to the formation of a viscoelastic material. Thermal energy (TE) may be introduced by additional steam increasing the temperature, ranging from 125 to 150 °C but also from the mechanical energy (ME) dissipated from the rotating screw components. The moisture content usually reaches 23 to 28% before exiting the extruder through (4) the die. The die’s restricted diameter increases shearing and pressure, leading to an instant pressure drop and the creation of an aerated structure in the extruded food once at atmospheric pressure. The resulting moist kibbles are then (5) dried, cooled and (6) coated before being (7) packaged [17,18,178]. Extrusion cooking is an extreme process that leads to ingredient physicochemical transformations that significantly impact palatability either positively or negatively.

Koppel et al. [109] focused on thermal-to-mechanical energy ratio effects on sensory attributes and concluded that a higher ratio resulted in lighter, more porous, and less hard kibbles. This is consistent with an increase in starch gelatinization due to an increased steam ratio [17]. Lower mechanical energy inputs also led to less macromolecular degradation of the dough structure [109]. Mechanical and thermal energies appear to be closely linked to the texture of materials. Indeed, while [109] stated a higher expansion with a high TE and low ME, Corsato Alvarenga et al. [178] reported reduced expansion with a lower ME but an unaltered TE. Pacheco et al. [179] increased the TE by increasing steam input in the preconditioner and decreased the ME by reducing screw speed in the extruder for a similar result on texturization. Thus, both parameters should be considered when specific texturization is being researched. However, while this resulted in higher expansion and lower hardness in [179], it did not impact palatability when tested in two-bowl tests with 36 dogs. Additionally, in [179], the authors managed to demonstrate a cost-effective production pattern without compromising palatability. Increasing both the TE and flow rate while decreasing the ME (screw speed) increased productivity with the same electric energy consumption. Similar results have been demonstrated for cat kibble production in terms of kibble structure and energy efficiency [180]. This time, palatability tests resulted in a higher intake ratio for an increase in the TE/ME ratio when tested on a panel of 20 cats. However, the moisture content between the two extreme variables decreased by 10 g/kg. Cats are known to prefer drier kibbles, where dogs prefer moister kibbles [148,155], which may have induced a preference in cats in the study [180].

In addition to the extrusion process, the ingredients used also impact process parameters. After starch, fats play a significant role in product texturization due to their lubricating properties [142,145]. Adding fat or ingredients rich in lipids decreases in-barrel temperature, pressure, and ME, while fiber may differently impact mechanical energy depending on its sources, impacting product expansion [134,142,145].

The production process of palatants, which are used to enhance palatability, is also crucial. Vanacker and Pibarot [181] disclosed a methodology to produce a liquid animal digest (LAD) at high pH instead of traditionally low pH (below pH 3.5). Increasing the pH to at least 9.5 prevented Salmonella growth as well as a low pH and significantly improved the palatability of dry dog food compared to kibble coated with a low pH LAD.

The application technique of coatings has also been studied to improve palatability. Proper sequencing of fat and palatant layers, allowing for proper distribution and homogenous coating among a batch of kibbles, has been found to enhance palatability [154]. Indeed, applying fat first followed by the palatant (liquid or powder) positively impacted the first choice and consumption ratio against kibbles coated with a mix of fat and palatant when tested on 36 dogs [154].

Alternative coating applications have also been studied, such as vacuum coating to offer better fat inclusion through the pores of the kibble [182,183,184]. Mainly considered for feeds that would need an important level of fat in the coating, such as trout feed [183], vacuum coating may be useful when kibbles are coated with powder palatants to prevent them from being embedded in the fat. Revéret and Bramoullé [184] compared vacuum coating and differential cooling (coating powder 24 h after fat) to standard coating on powder availability to cats on dry food. Three levels of fat coating were considered: 8%, 11% and 14%. The differential cooling and vacuum coating offered significantly better powder availability than regular coating, highlighting its ability in preventing fat from covering the palatant. The variables were fed in two-bowl tests to 40 cats. Differential cooling was revealed to be significantly more palatable than regular coating at all fat levels and vacuum coating only at the highest level. This demonstrated the potential to improve the palatability of high-topical fat products.

## 6. Conclusions

Despite the convenience of usage and storage, extruded dry pet food suffers from lower palatability than moist food. Consequently, understanding the mechanisms of food selection by pets and the various factors influencing it is essential to ensure that cats and dogs meet their daily nutritional requirements by consuming their meals.

Indeed, managing to produce a palatable product is of high importance for pets to feed themselves and even more for those with health issues. Palatability also appears as a main criterion for pet owners when considering buying pet food. Therefore, common methods exist to evaluate the acceptability and preference of a pet food by presenting respectively one or two products at a time to the pets. Interpreting the reasons for different acceptance or preference of a food towards another can be intricate due to both the product complexity multiplying potential influencing factors and the fact of working with nonspeaking living beings. For those matters, these tests can be completed by behavioral studies or human sensory analyses. They will give insights on potential reasons for rejection (respectively attraction) of a product or on the impact of process, recipe, and product characteristics on their organoleptic characteristics. Moreover, an extensive understanding of physiological, behavioral, and external factors is essential to understanding animals’ reactions to products. Looking back at cats’ and dogs’ ancestors and at their actual wild counterparts provides precious insights explaining their behaviors towards food. Evolution and adaptation to their specific diet and to domestication through time led to behavioral changes and specialization of their senses, making them selectively sensitive to some stimuli. Finally, the choice of raw materials and process parameters greatly influence the organoleptic properties of end products. The complexity of the formulation of the core kibble and of its coating, serving nutritional needs and palatability, and the quite extreme manufacturing process are leading to various modifications, reactions, and interactions of the product components. The growing environmental concerns in terms of energy efficiency and raw materials supply open both new challenges and opportunities, slowly introducing new pet food containing algae, mushrooms, or insect-based ingredients with satisfying palatability. These topics are also a concern for pet owners, for whom pets become members of the family, and ask for more humanized pet food, including “natural”, “raw” and “organic” food [185].

## Figures and Tables

**Table 1:** Main pros and cons of the different range of pet food depending on moisture level plus insights on homemade pet food.

	Dry	Semimoist	Wet	Homemade
**Moisture range**	<14%	14% to 60%	>60%	Variable
**Pros**	Storage	Storage	Source of dietary water and hydration	Enjoyable for both owners and pets
	Ease of use	Ease of use	Greatest palatability	Higher sense of involvement in pets’ health for owners
	No spoilage enabling free choice feeding	Higher palatability than dryer pet food	High level of proteins on dry-matter basis	Feeling for owners to better understand the ingredients used
	Dental health	-	Easier to chew (when dental issues)	-
	Cheapest on cost-per-calorie basis	-	-	-
**Cons**	Lower palatability than moister pet food	Readily available monosaccharides (diabetic pets)	Most expensive on cost-per-calorie basis	Often nutritionally inadequate and deficient
	-	-	-	Recipes not always properly observed by owners
	-	-	-	More expensive than dry products on cost-per-calorie basis
**References**	[5,28,29]	[5]	[5,28,29]	[29,30,31]

## Data Availability

Not applicable.
